# Performances of acute kidney injury biomarkers vary according to sex

**DOI:** 10.1093/ckj/sfae091

**Published:** 2024-03-27

**Authors:** Stanislas Faguer, Alexis Piedrafita, Ana Belen Sanz, Justyna Siwy, Ioanna K Mina, Melinda Alves, Paul Bousquet, Bertrand Marcheix, Audrey Casemayou, Julie Klein, Vincent Minville, Benjamin Breuil, Alberto Ortiz, Joost P Schanstra

**Affiliations:** Department of Nephrology and Organ Transplantation, French Intensive Care Renal Network, University Hospital of Toulouse, Toulouse, France; National Institute of Health and Medical Research, UMR 1297 (Institute of Metabolic and Cardiovascular Diseases), Toulouse, France; Faculty of Health, University Paul Sabatier – Toulouse-III, Toulouse, France; Department of Nephrology and Organ Transplantation, French Intensive Care Renal Network, University Hospital of Toulouse, Toulouse, France; National Institute of Health and Medical Research, UMR 1297 (Institute of Metabolic and Cardiovascular Diseases), Toulouse, France; Faculty of Health, University Paul Sabatier – Toulouse-III, Toulouse, France; IIS-Fundación Jiménez Díaz, School of Medicine, Autonomous University of Madrid, FRIAT and RICORS2040, Madrid, Spain; Mosaiques Diagnostics GmbH, Hannover, Germany; Mosaiques Diagnostics GmbH, Hannover, Germany; Institute for Molecular Cardiovascular Research, RWTH Aachen University Hospital, Aachen, Germany; National Institute of Health and Medical Research, UMR 1297 (Institute of Metabolic and Cardiovascular Diseases), Toulouse, France; Faculty of Health, University Paul Sabatier – Toulouse-III, Toulouse, France; Department of Anesthesiology and Critical Care, University Hospital of Toulouse, Toulouse, France; Faculty of Health, University Paul Sabatier – Toulouse-III, Toulouse, France; Department of Cardiac and Vascular Surgery, University Hospital of Toulouse, Toulouse, France; Department of Nephrology and Organ Transplantation, French Intensive Care Renal Network, University Hospital of Toulouse, Toulouse, France; National Institute of Health and Medical Research, UMR 1297 (Institute of Metabolic and Cardiovascular Diseases), Toulouse, France; Faculty of Health, University Paul Sabatier – Toulouse-III, Toulouse, France; National Institute of Health and Medical Research, UMR 1297 (Institute of Metabolic and Cardiovascular Diseases), Toulouse, France; Faculty of Health, University Paul Sabatier – Toulouse-III, Toulouse, France; Faculty of Health, University Paul Sabatier – Toulouse-III, Toulouse, France; Department of Anesthesiology and Critical Care, University Hospital of Toulouse, Toulouse, France; National Institute of Health and Medical Research, UMR 1297 (Institute of Metabolic and Cardiovascular Diseases), Toulouse, France; Faculty of Health, University Paul Sabatier – Toulouse-III, Toulouse, France; IIS-Fundación Jiménez Díaz, School of Medicine, Autonomous University of Madrid, FRIAT and RICORS2040, Madrid, Spain; National Institute of Health and Medical Research, UMR 1297 (Institute of Metabolic and Cardiovascular Diseases), Toulouse, France; Faculty of Health, University Paul Sabatier – Toulouse-III, Toulouse, France

**Keywords:** acute kidney injury, biomarkers, NephroCheck, NGAL, peptidomics, sexual dimorphism

## Abstract

**Background:**

Before implementing individualized strategies to treat acute kidney injury (AKI), identifying clusters of patients with divergent pathophysiological mechanisms, diagnosis criteria or outcomes is of the utmost importance. Here we studied sex-related molecular mechanisms in cardiac bypass (CBP) surgery patients developing AKI.

**Methods:**

We compared the characteristics of 1170 patients referred for CBP surgery using multivariate logistic regression and propensity score–based analysis. Performances of the candidate urinary biomarkers at <4 h post-surgery, urinary neutrophil gelatinase-associated lipocalin (uNGAL), [IGFBP7]·[TIMP-2] product (NephroCheck) and a recently developed AKI signature of 204 urinary peptides (AKI204) to predict AKI were compared in both sexes.

**Results:**

Incidence (∼25%) and severity of AKI were similar in men and women, even after adjustment for the usual risk factors of AKI, including baseline estimated glomerular filtration rate, age, diabetes mellitus, length of CBP and red blood cell transfusion. However, at the molecular level, performances of uNGAL, NephroCheck and AKI204 to predict AKI strongly diverged between men and women. In the full cohort, as well as in subgroups of men and women, the multimarker AKI204 signature outperformed uNGAL and NephroCheck and predicted the development of AKI significantly better in women than in men. Analysis of AKI204 at the single-peptide level suggested divergences of AKI mechanisms between sexes due to increased kidney inflammation in women (increased abundance of urinary fragments of osteopontin and uromodulin).

**Conclusions:**

In patients referred for CBP surgery, significant clinical and biological differences between men and women as well as sexual dimorphism of AKI biomarker performances were identified. The urinary peptide signature points to sex-related molecular mechanisms underlying AKI.

KEY LEARNING POINTS **What was known:**

- Acute kidney injury (AKI) is a prototypical condition with sexual dimorphism in animals, but data remain elusive in humans.
- The absence of sex-disaggregated data in AKI studies may have led to false conclusions regarding stratification and treatment effects in subgroups, ruling out specific risk factors.
- Whether performances of urinary biomarkers are similar in both males and females remains elusive.

**This study adds:**

- In a series of 1170 individuals referred for CBP surgery, the incidence of AKI was similar in men and women (∼25%) even after adjustment for the usual risk factors of AKI.
- However, at the molecular level, performances of urinary neutrophil gelatinase-associated lipocalin (uNGAL), [IGFBP7]·[TIMP-2] (NephroCheck) and an AKI signature of 204 urinary peptides (AKI204) to predict AKI strongly diverged between men and women, with the multimarker AKI204 signature outperforming uNGAL and NephroCheck and predicting the development of AKI significantly better in women than in men.
- Analysis of AKI204 at the single-peptide level suggested divergences of AKI mechanisms between sexes due to increased kidney inflammation in women.

**Potential impact:**

- A similar level of AKI severity does not exclude sex-related divergent molecular mechanisms of AKI, suggesting that a reappraisal of the AKI definition may help to personalize AKI diagnosis and develop a mechanistic-driven AKI management.
- The ability of the multimarker peptide AKI204 signature to integrate data issued from different kidney compartments and potentially the circulation probably underlies its global better performances compared with single biomarkers of tubular stress/injury.
- These results confirmed the potential of a urinary peptide signature to develop personalized medicine for AKI by giving quantitative assessment of the risk to develop AKI after a kidney attack, but also to give qualitative information underlying molecular patterns of AKI.

## INTRODUCTION

There is cumulative evidence that sex dramatically influences the course of acquired diseases, including autoimmune diseases, cardiovascular disorders and cancer. Acute kidney injury (AKI) is a prototypical condition with sexual dimorphism in animals, as most ischaemic or toxic kidney insults induce severe AKI in males but have no or mild to moderate consequences in females [1–3]. At the transcriptional level, early kidney changes following ischaemic AKI in mice strongly diverge between males and females [4].

Among others, mechanisms of resistance to kidney injury involve differential expression of sex hormone–dependent genes in immune and tubular cells, with different sensitivity or response of these cells to injury [5, 6]. In humans, data are more conflicting, but a recent meta-analysis confirmed the protection offered by female sex against AKI in a non-cardiac surgery setting [7]. Such discrepancies between studies may have emerged from a lack of robustness of the endpoint criteria between sexes (e.g. variability of the serum creatinine production rate between males and females, divergences of maximal serum creatinine reached between sexes), the underlying sexual dimorphisms of kidney resistance to injury and male and female heterogeneity of the acute condition leading to AKI. In addition, the absence of sex-disaggregated data in AKI studies may have led to false conclusions regarding stratification and treatment effects in subgroups, ruling out specific risk factors and thus slowing down research progress in the AKI field.

In this study, we took advantage of a large cohort of 1170 patients subjected to cardiac bypass (CBP) surgery to estimate and compare the incidence and risk factors of AKI in males and females as well as the performance of candidate AKI risk urinary biomarkers [neutrophil gelatinase-associated lipocalin (NGAL), [IGFBP7]·[TIMP-2] product (NephroCheck) and a unique 204 urinary peptides signature (AKI204)] [8] in this prototypic high-risk situation of AKI.

## MATERIALS AND METHODS

### Cohorts

The cohort included in this study has been previously described (see Piedrafita *et al*. [8]). Patients referred for CBP surgery were prospectively recruited at the University Hospital of Toulouse (France) during two distinct time periods [March 2016–January 2017 (*n* = 509) and January 2019–March 2020 (*n* = 661)]. Patients <18 years of age who underwent unscheduled CBP surgery, who required chronic dialysis before surgery or who had ongoing AKI at the time of the surgery were excluded. All the patients were orally informed of their inclusion during anaesthetic consultation performed in the weeks before surgery and agreement of the patients to be included in the clinical and biological collection of the University Hospital of Toulouse was obtained before inclusion (French national ethical committee agreement number DC-2008-463). The study was performed according to the Declaration of Helsinki as revised in 2004.

### Clinical data

Pre-, peri- and postoperative clinical data were gathered retrospectively for all patients based on hospital records. Baseline estimated glomerular filtration rate (eGFR) was determined using the Chronic Kidney Disease Epidemiology Collaboration (CKD-EPI) formula based on standardized creatinine measurement (isotope dilution mass spectrometry) before cardiac surgery. European System for Cardiac Operative Risk Evaluation II (EuroSCORE II) was calculated as recommended (https://euroscore.org/).

Surgery was divided into coronary artery bypass (CAB), valvular surgery (valvuloplasty or replacement), combined CAB and valvular surgery, surgery with replacement of the ascending aorta with or without CAB (aortic surgery) and surgery that directly affects the cardiac myocardial wall, such as interatrial communication, interventricular communication, ventricular aneurism or cardiac transplantation (myocardium).

### Definitions

The main endpoint, AKI, was defined according to the Kidney Disease: Improving Global Outcomes (KDIGO) 2012 criteria evaluated during the first 7 days after surgery [9]. AKI was defined as a significant increase in serum creatinine (>1.5 times baseline or >26.5 μmol/l increase) or a decreased urine output (<0.5 ml/kg/h for at least 6 h) or renal replacement therapy (RRT) requirement.

Pulmonary hypertension was defined as systolic pulmonary arterial pressure >30 mmHg after depletion (cardiac echography).

### Biomarker measurement

Urine samples were collected 2.5–4 h after the end of cardiac bypass and immediately frozen (−20°C) before subsampling and refrozen for long-term conservation (−80°C). Urinary peptidome analysis was performed as previously described [8]. For urinary neutrophil gelatinase-associated lipocalin (uNGAL) measurement, urine samples were centrifuged for 10 min at 2500 rpm. NGAL was measured using an enzyme-linked immunosorbent assay (Human Lipocalin-2/NGAL Duoset ELISA; DY1757; R&D Systems, Minneapolis, MN, USA) in diluted supernatant (1:10 or 1:100) according to the manufacturer's protocol. Creatinine was measured using the QuantiChrom Creatinine Assay Kit (DICT 500; BioAssay Systems, Hayward, CA, USA) according to the manufacturer's protocol. Creatinine-normalized uNGAL (μg/g) was used for performance evaluations. [TIMP-2] and [IGFBP7] were measured in urine supernatants using the VITROS NephroCheck immunoassay on a VITROS 5600 Integrated System (Ortho Clinical Diagnostics, Raritan, NJ, USA) according to the manufacturer's instructions. The VITROS NephroCheck test result is a single number, which is a product of the measured concentrations of the two analytes in the sample divided by 1000. Certified laboratory technicians blinded to clinical data performed the analyses.

### Statistical analyses

For clinical descriptive analysis, missing data were removed [left ventricular ejection fraction (LVEF); *n* = 2 missing data) by pairwise deletion. Quantitative variables were presented as mean ± standard deviation (SD) and qualitative variables as number and percentage. Two-group comparisons were conducted using the Mann–Whitney (continuous variables), χ^2^ or Fisher's exact (discontinuous variables) tests, as appropriate. Multivariable analysis was based on logistic regression and includes all preoperative variables with univariate adjusted *P*-values <.1. The model qualities of the logistic regressions were verified with the Hosmer–Lemeshow test. To identify peptides differentially represented in urines according to AKI and sex status, a two-way analysis of variance corrected for the false discovery rate using the Benjamini–Hochberg procedure was used. *P*-values <.05 were considered significant. All analyses were performed with the XLSTAT software (Addinsoft, Paris, France).

## RESULTS

### Sex differences at referral for cardiac surgery

Among the 1170 CBP surgery individuals of the cohort, 288 were women (24.6%) (Table 1). Univariate analysis showed that women referred for CBP surgery were older, had a lower body mass index (BMI) and less frequently had diabetes mellitus and peripheral vascular disease but more frequently had pulmonary arterial hypertension compared with men. Pre-surgery eGFR was lower (72 ± 23 versus 75 ± 20 ml/min/1.73 m^2^, *P* = .03) while LVEF was higher (59 ± 9% versus 55 ± 11%; *P* < .0001) in women.

**Table 1:** Clinical characteristics according to sex.

Parameters	All patients (*n* = 1170)	Females (*n* = 288)	Males (*n* = 882)	*P*-value
Preoperative features				
Age (years), mean ± SD	66.7 ± 12	68 ± 14	66 ± 11	<.001
BMI (kg/m^2^), mean ± SD	27 ± 4.6	26 ± 6	27 ± 4	<.0001
Diabetes, n (%)	306 (26)	62 (22)	244 (28)	.04
Hypertension, n (%)	668 (57)	166 (58)	502 (58)	.83
PVD, n (%)	108 (9)	14 (5)	94 (11)	<.001
COPD, n (%)	126 (11)	35 (12)	91 (10)	.39
PAH, n (%)	157 (13.4	51 (18)	106 (12)	.02
EuroSCORE II, mean ± SD	2.6 ± 3.6	3.2 ± 3.4	2.4 ± 3.7	<.0001
LVEF (%), mean ± SD	56 ± 11	59 ± 9	55 ± 11	<.0001
Serum creatinine (μmol/l), mean ± SD	93 ± 36	80 ± 34	97 ± 35	<.0001
eGFR (ml/min.1.73 m^2^), mean ± SD	74 ± 21	72 ± 23	75 ± 20	.03
Kidney graft recipients, n (%)	13 (1)	5 (2)	8 (1)	.27
Perioperative features				
Surgery, *n* (%)				<.0001
CAB	441 (38)	67 (23)	374 (42)	
Valvular	374 (32)	137 (48)	237 (27)	
Combined	176 (15)	30 (10)	146 (17)	
Thoracic aorta	145 (12)	37 (13)	108 (12)	
Myocardium	34 (3)	17 (6)	17 (2)	
Previous cardiac surgery, *n* (%)	80 (7)	30 (10)	50 (6)	<.01
CBP time (min), mean ± SD	87 ± 36	81 ± 40	89 ± 35	<.0001
RBC transfusion, *n* (%)	150 (13)	74 (26)	76 (9)	<.0001
Mean ± SD	0.3 ± 0.9	0.6 ± 1.2	0.2 ± 0.7	<.0001
Vasoactive agents, *n* (%)	1083 (93)	272 (94)	811 (92)	.15
Postoperative features				
SAPS II score, mean ± SD	33 ± 12	34 ± 12	32 ± 11	<.01
RBC transfusion, *n* (%)	306 (26)	100 (35)	206 (23)	<.001
Mean ± SD	0.7 ± 1.8	0.9 ± 2.1	0.7 ± 1.7	.001
Vasoactive agents, n (%)	727 (62)	181 (63)	541 (61)	.65
CVP (mmHg), mean ± SD	12.3 ± 5.5	12.3 ± 7.4	12.3 ± 4.8	.48
Infection, *n* (%)	195 (17)	34 (12)	161 (18)	<.01
Iodinated contrast agents, *n* (%)	32 (3)	8 (3)	24 (3)	.96
Fluid infusion day 1				
ml, mean ± SD	1182 ± 716	1205 ± 713	1174 ± 718	.53
ml/kg, mean ± SD	16 ± 11	19 ± 13	15 ± 10	<.0001
ICU stay (days), mean ± SD	4.8 ± 6	4.7 ± 4.7	4.8 ± 6.3	.29
Outcomes				
AKI KDIGO stage, *n* (%)				.99
0	875 (74)	214 (74)	661 (75)	
1	160 (14)	39 (14)	121 (14)	
2	92 (8)	24 (8)	68 (8)	
3	43 (4)	11 (4)	32 (4)	
RRT, *n* (%)	32 (3)	10 (3)	22 (2)	.52
In-hospital mortality, *n* (%)	38 (3)	13 (4.5)	25 (2.8)	.18

COPD: chronic obstructive pulmonary disease; CVP, central venous pressure; PVD: peripheral vascular disease; PAH: pulmonary arterial hypertension.

Indications for cardiac surgery were significantly different, with valvular-only surgery more frequent in women but CAB or combined valvular–CAB surgery were more frequent in men (*P* < .0001). More women were also referred for a second or further cardiac surgery. This resulted in a significantly higher EuroSCORE II mortality risk in women (3.2 ± 3.4 versus 2.4 ± 3.7; *P* < .0001).

### Sex differences in peri- and postoperative management

During surgery, women more frequently received red blood cells (RBCs) despite a shorter length of cardiac bypass (Table 1).

At admission to the intensive care unit (ICU), the Simplified Acute Physiology Score II (SAPS II) gravity score was higher (*P* < .01) and postoperative transfusion more frequent (*P* < .001). The volume of fluid infusion during the first postoperative day was higher in women (19 ± 13 versus 15 ± 10 ml/kg; *P* < .0001). However, the use of vasopressive agents as well as the maximal central venous pressure were similar between the two groups.

### Incidence and severity of AKI according to sex

Univariate analysis showed that the incidence and severity of AKI in the CBP surgery cohort was similar between men [221/882 (25%)] and women [74/288 (26%)], as well as the use of postoperative RRT and in-hospital mortality at day 30.

Multivariable analysis (logistic regression–based analysis including pre- and perioperative variables that discriminated male and female individuals and patients developing or not developing AKI) indicated that sex was not associated with the risk of developing AKI. To confirm this result, a propensity score–based multivariable analysis was performed and did not identify differences between male and female patients in the risk of developing AKI.

### Predictive factors of AKI according to sex

In the next step, we aimed to identify predictive factors of AKI in each sex subgroup. As shown in Table 2, univariate analysis identified common (age, furosemide use before surgery, EuroSCORE II, pre-surgery eGFR, CBP time and RBC transfusion) but also divergent (BMI, hypertension, LVEF, indication for surgery, previous cardiac surgery) predictive factors. The number of predictive factors associated with the risk of developing AKI was higher in men.

**Table 2:** Risk factors for AKI in men and women.

	Women			Men		
Parameters	No AKI (n = 214)	AKI (n = 74)	*P*-value*	No AKI (n = 661)	AKI (n = 221)	*P*-value^#^
Preoperative features						
Age (years), mean ± SD	66 ± 15	72 ± 8	.02	65 ± 12	69 ± 10	<.001
BMI (kg/m^2^), mean ± SD	26 ± 5	27 ± 6	.23	26.7 ± 4	28.4 ± 5	<.0001
Diabetes, *n* (%)	41 (19)	21 (28)	.10	172 (26)	72 (33)	.07
Hypertension, *n* (%)	117 (55)	49 (66)	.10	347 (53)	155 (70)	<.0001
PVD, *n* (%)	8 (4)	6 (8)	.15	66 (10)	28 (13)	.27
COPD, *n* (%)	22 (10)	13 (18)	.11	64 (10)	27 (12)	.29
PAH, *n* (%)	33 (15)	18 (24)	.11	71 (11)	35 (16)	.05
Furosemide use, *n* (%)	55 (26)	32 (43)	.005	131 (20)	88 (40)	<.0001
SAPS II score, mean ± SD	32 ± 9	41 ± 16	<.0001	30 ± 9	38 ± 14	<.0001
EuroSCORE II, mean ± SD	2.3 ± 2	5.9 ± 5	<.0001	1.9 ± 3	3.6 ± 5	<.0001
LVEF (%), mean ± SD	59 ± 9	59 ± 10	.85	56 ± 11	53 ± 13	.03
Serum creatinine (μmol/l), mean ± SD	74 ± 21	98 ± 53	<.0001	93 ± 24	110 56	<.001
eGFR (ml/min/1.73 m^2^), mean ± SD	76 ± 21	61 ± 23	<.0001	77 ± 18	68 ± 24	<.0001
Kidney graft recipients, n (%)	2 (1)	3 (4)	.10	2 (0.3)	6 (3)	.003
Perioperative features						
Surgery, *n* (%)			.11			<.001
CAB	55 (26)	12 (16)		303 (46)	71 (32)	
Valvular	103 (48)	34 (46)		181 (27)	56 (25)	
Combined	18 (8)	12 (16)		93 (14)	53 (24)	
Thoracic aorta	24 (11)	13 (18)		73 (11)	35 (16)	
Myocardium	14 (7)	3 (4)		11 (2)	6 (3)	
Previous cardiac surgery, *n* (%)	18 (8)	12 (16)	.07	27 (4)	23 (10)	.001
CBP time (min), mean ± SD	75 ± 33	100 ± 51	<.0001	85 ± 31	102 ± 43	<.0001
RBC transfusion, n (%)	47 (22)	27 (36)	.02	40 (6)	36 (16)	<.0001
Mean ± SD	0.5 ± 0.9	1.0 ± 1.7	.007	0.1 ± 0.2	0.2 ± 0.4	<.0001
Postoperative features						
RBC transfusion, *n* (%)	57 (27)	43 (58)	<0.0001	114 (17)	92 (42)	<.0001
Mean ± SD	0.6 ± 1	1.9 ± 3.6	<0.0001	0.4 ± 1	1.5 ± 3	<.0001
Vasoactive agents, *n* (%)	119 (56)	62 (84)	<0.0001	376 (57)	165 (75)	<.0001
CVP (mmHg), mean ± SD	12 ± 8	13 ± 5	0.03	12 ± 5	13 ± 5	.001
Infection, *n* (%)	15 (7)	19 (26)	<0.0001	88 (13)	73 (33)	<.0001
Iodinated contrast agents, *n* (%)	5 (2)	3 (4)	0.45	9 (1)	15 (7)	<.0001
Fluid infusion day 1 (ml), mean ± SD	1106 ± 673	1490 ± 751	<0.0001	1111 ± 695	1362 ± 750	<.0001
ICU stay (days), mean ± SD	4 ± 3	7 ± 7	<0.0001	4 ± 4	8 ± 10	<.0001
Mortality at day 30, *n* (%)	2 (0.9)	10 (13.5)	<0.0001	4 (0.6)	14 (6.3)	<.0001

*P-values of statistical analyses performed in women and ^#^*P*-values of statistical analyses performed in men. COPD: chronic obstructive pulmonary disease; CVP: central venous pressure; PVD: peripheral vascular disease; PAH: pulmonary arterial hypertension.

By multivariable analysis using logistic regression, pre-surgery eGFR and the length of CBP were the only predictive factors of AKI in women. In contrast, in men, age, BMI, pre-surgery eGFR, length of CBP, a history of previous cardiac surgery, hypertension, kidney transplantation and preoperative use of furosemide were independently associated with the risk of developing AKI after CBP surgery (Table 3).

**Table 3:** Predictive factors for AKI after CBP surgery in female and male patients (multivariable analysis using logistic regression).

	*P*-value	Odds ratio	95% CI
Risk factors for CBP AKI in women			
eGFR (CKD-EPI)	<.0001	0.97	0.95–0.98
Length of CBP	<.0001	1.02	1.01–1.03
Risk factors for CBP AKI in men			
Age	.010	1.02	1.01–1.04
BMI	<.0001	1.10	1.05–1.14
eGFR (CKD-EPI)	.013	0.99	0.98–0.99
Length of CBP	<.0001	1.01	1.01–1.02
Previous cardiac surgery	.005	2.67	1.35–5.27
Hypertension	.002	1.77	1.23–2.57
Kidney recipient	.003	15.9	2.9–130
Preoperative use of furosemide	<.0001	2.26	1.56–3.26

### Performance of urinary AKI biomarkers according to sex

**Figure 1:**
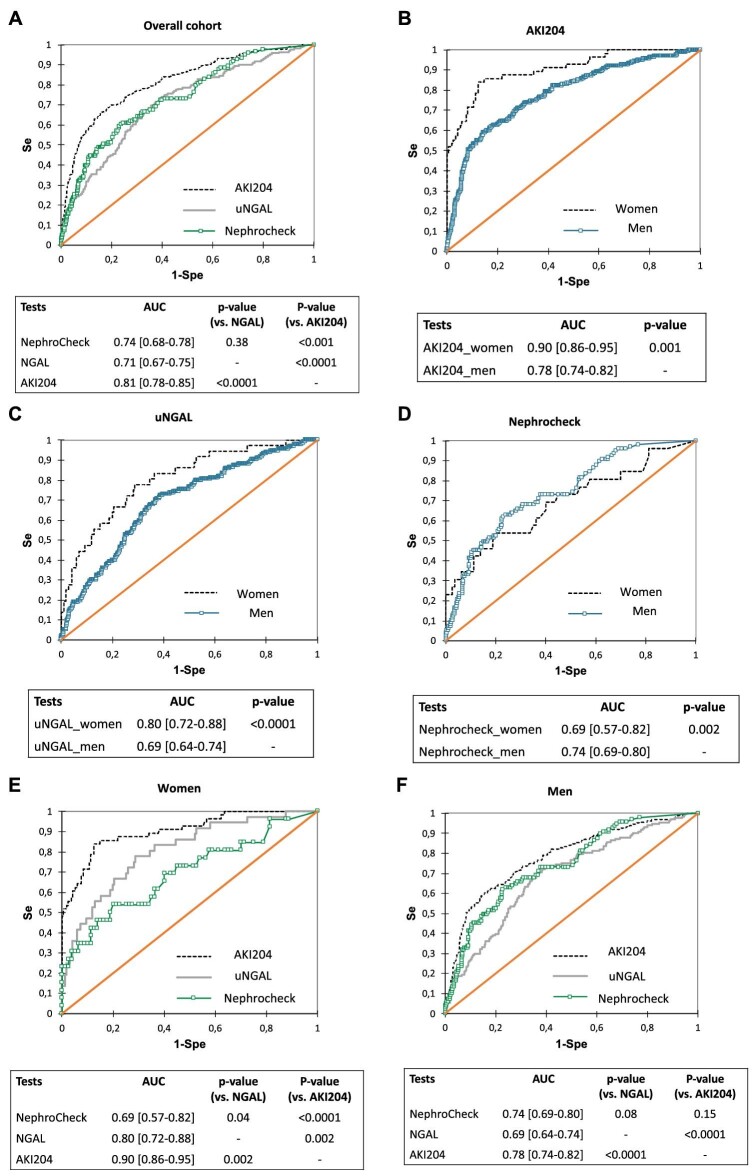
Performances of urinary biomarkers to predict AKI in a cohort of CBP surgery patients (receiver operating characteristics curves analysis). **(A)** Overall cohort, all biomarkers (AKI204 versus uNGAL, *P* < .001; AKI204 versus NephroCheck, *P* < .001; uNGAL versus NephroCheck, *P* = .38. Men and women assessed separately for **(B)** AKI204 (*P* < .001), **(C)** NephroCheck (*P* = .002) and **(D)** uNGAL (*P* < .001). **(E)** Men, all biomarkers (AKI204 versus uNGAL, *P* < .001; AKI204 versus NephroCheck, *P* = .15; uNGAL versus NephroCheck, *P* = .08). **(F)** Women, all biomarkers (AKI204 versus uNGAL, *P* = .002; AKI204 versus NephroCheck, *P* < .001; uNGAL versus NephroCheck, *P* = .04).

Values for uNGAL, NephroCheck and the AKI204 peptide signature in urine collected ≤4 h after the end of the CBP surgery were available in 856, 462 and 926 patients, respectively. The performance of the AKI204 signature to predict AKI in the overall cohort was significantly higher than that of uNGAL and NephroCheck (*P* < .0001 and *P* < .001, respectively; Fig. 1A). When analysed separately, the performance of each biomarker varied according to sex: performances of AKI204 and uNGAL were significantly better in women compared with men (Fig. 1B and C), whereas those of NephroCheck were better in men (Fig. 1D).

When the predictive performance of biomarkers of AKI was compared in men and women, the multimarker AKI204 was significantly superior to both uNGAL and NephroCheck in women (Fig. 1E) and superior to uNGAL only in men (Fig. 1F). Predictive performances of AKI biomarkers in men and women after adjustment for age, baseline eGFR, history of cardiac surgery, CBP duration and RBC transfusion (multivariable logistic regression) confirm these findings (Supplementary Table S1).

### Urinary peptide signature points to sex-divergent AKI mechanisms

**Figure 2:**
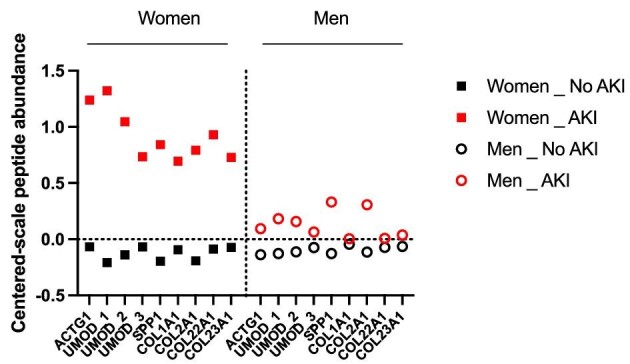
Urinary peptides 3–4 h after CBP surgery that are significantly divergent in women or men developing AKI among the 204 peptides of AKI204. Peptides with *P*-values <.05 (after Benjamin–Hochberg correction for multiple testing) are reported (mixed effects analysis of AKI and gender).

Because performances of the AKI204 strongly diverged between males and females, we hypothesized that individual changes in the 204 peptides may inform on the sexually divergent mechanisms underlying AKI (i.e. a similar quantitative score predictive of AKI may rely on different cluster-based peptide variations according to sex). Indeed, the abundance of a number of individual urinary peptides between AKI and non-AKI patients varied according to sex. In women developing AKI, in addition to a number of collagen fragments, urinary peptides derived from inflammatory proteins (SPP1/osteopontin) or reflected pro-inflammatory kidney-specific changes (uromodulin/Tamm–Horsfall protein) were significantly more abundant than in men (Fig. 2).

## DISCUSSION

Before implementing individualized strategies to prevent or treat AKI, identifying clusters of patients with common (or divergent) pathophysiological mechanisms, diagnosis criteria or outcomes is of the utmost importance. In our series of 1170 individuals referred for CBP surgery, the incidence of AKI was similar in men and women (≈25%) even after adjustment using the usual risk factors of AKI, including baseline eGFR, age, diabetes mellitus, length of CBP and RBC transfusion. These findings are consistent with the results of a recent meta-analysis that included ≈7 million individuals and reported a similar incidence of AKI between the sexes when analyses are restricted to patients referred for CBP surgery [7]. Because most women referred for CBP surgery are mostly menopausal, the discrepancies between epidemiological studies in human and pre-clinical models of AKI may rely on methodological pitfalls in animal studies that mostly include young (male) mice, as already previously described [10].

Importantly, the incidence of AKI was similar in both sexes in our cohort, but clinical characteristics at referral, as well as performance of urinary AKI biomarkers, significantly diverged between women and men, pointing to potential divergent molecular mechanisms of AKI.

The difference in prediction efficacies of uNGAL, AKI204 and NephroCheck in men and women could be due to a combination of differences in AKI pathophysiology in men and women and the origin of the measured markers in different renal and vascular compartments. NGAL is expressed by both proximal and distal tubules, as well as in the collecting duct [11, 12], and is also filtered from the blood. IGFBP7 and TIMP2 (i.e. NephroCheck) are derived from the proximal tubule [13]. In contrast, urinary peptides are derived from (filtered) plasma and from all nephron segments.

In parallel, recent data obtained in a pig model of ischaemic AKI showed that similar levels of renal dysfunction (as assessed by measurement of blood urea nitrogen and serum creatinine) were underpinned by different clear sex-dependent molecular mechanisms [4]. After ischaemia–reperfusion injury, kidneys of female pigs were characterized by higher mononuclear infiltrates, whereas kidneys of male pigs were characterized by more severe tubular injury. Kidney expression of genes involved in immune and inflammatory processes were also significantly higher in female kidneys at basal conditions and during injury. We also observed a shift to increased urinary abundance of inflammation-related peptides (osteopontin/SPP1 and Tamm–Horsfall protein/uromodulin) in woman developing AKI compared with men. In addition, it is also likely that some of the uNGAL is not specifically kidney derived but is produced by neutrophils in plasma (i.e. inflammatory responses) and ends up in urine due to its relatively small size (25 kDa) and kidney sieving dysfunction during AKI. Higher inflammation in women may thus improve its prognostic value in women compared with men. In contrast, kidneys of male pigs were characterized by more severe tubular injury, which could explain the optimal performance of NephroCheck in the male population. Thus, while the AKI peptide signature was not designed to identify relevant pathophysiological mechanisms of AKI, this may suggest that AKI should not be seen only as an abrupt decrease or loss of excretory kidney function, but as a complex acute disorder [14] that includes diverse pathophysiological mechanisms, potentially different between sexes. A similar level of AKI severity does not exclude sex-related divergent molecular mechanisms of AKI, suggesting that a reappraisal of the AKI definition may help to personalize the AKI diagnosis and develop mechanistic-driven AKI management. Our results also strongly advocate for always taking sex into consideration when developing new biomarkers or treatments for AKI and reanalysing biomarker literature data in light of the male:female ratio of individuals included in studies.

Beyond direct molecular mechanisms of AKI, sexual divergences may also contribute to the history of AKI progression owing to socio-economic status and ethnicity, factors rarely included in epidemiological studies [15]. Environmental factors including dietary habits and genetic and epigenetic factors in some populations may also explain part of the divergences in studies performed in various countries. This was also demonstrated in mice models of AKI [16]. In our cohort, perioperative management and characteristics also differed between sexes, with more pre- and post-surgery RBC transfusions and more postoperative fluid loading in women, both of which are associated with AKI during cardiac surgery. Further studies will therefore need to decipher the respective roles of potential mechanistic divergences and differences in perioperative management between sexes.

Candidate single or low-resolution urinary biomarkers of AKI focusing on part of the AKI pathology, such as epithelial injury (NGAL, KIM-1), cell cycle arrest ([IGFBP7]·[TIMP-2] product; NephroCheck) or metabolism disorders (urinary quinolinate:tryptophan ratio, liver-type fatty acid-binding protein) of tubular cells, lack—by concept—the ability to grasp the full complexity of the mechanisms involved in AKI development (including immune and endothelial mechanisms) and its trajectories (i.e. the various molecular patterns that may underlie glomerular filtration reductions of similar intensity). Such single or low-resolution markers are therefore probably insufficient to reach true personalized medicine and individualize treatments. Here, extending our analysis to the overall cohort of 1170 patients, we showed that AKI204 outperformed both uNGAL and NephroCheck. The ability of the multimarker peptide signature to integrate data issued from different kidney compartments and potentially the circulation probably underlies its global better performance compared with single biomarkers of tubular stress/injury, including its higher sensitivity to detect AKI in women compared with conventional candidate biomarkers. If confirmed in further external cohorts, these results highlight the potential of a urinary peptide signature to develop personalized medicine for AKI by giving quantitative assessment of the risk to develop AKI after a kidney attack. It remains to be determined whether the urinary peptide signature will also provide qualitative insights into the underlying molecular patterns of AKI and potential actionable targets. These results may open new avenues in individualized treatment of AKI considering sex and prompt the testing of the ability of other non-invasive omics-based strategies to stratify patients according to their quantitative and qualitative risk of AKI, and beyond this to identify key AKI molecular hubs using systems biology applied to human cohorts.

## KIDNEY ATTACK STUDY GROUP

Stanislas Faguer, Department of Nephrology and Organ Transplantation, University Hospital of Toulouse, Toulouse, France; Bertrand Marcheix, Department of Cardiac and Vascular Surgery, University Hospital of Toulouse, Toulouse, France; Etienne Grunenwald, Department of Cardiac and Vascular Surgery, University Hospital of Toulouse, Toulouse, France; Vincent Minville, Department of Anesthesiology and Critical Care Medicine, University Hospital of Toulouse, Toulouse, France; Julia Grossac, Department of Anesthesiology and Critical Care Medicine, University Hospital of Toulouse, Toulouse, France; François Labaste, Department of Anesthesiology and Critical Care Medicine, University Hospital of Toulouse, Toulouse, France; Nicolas Mayeur, Department of Anesthesiology and Critical Care Medicine, University Hospital of Toulouse, Toulouse, France; Elsa Tardif, Department of Anesthesiology and Critical Care Medicine, University Hospital of Toulouse, Toulouse, France; Joost P. Schanstra, Institute for Metabolic and Cardiovascular Disease, National Institute of Health and Medical Research, Toulouse, France; Julie Klein, Institute for Metabolic and Cardiovascular Disease, National Institute of Health and Medical Research, Toulouse, France; Benjamin Breuil, Institute for Metabolic and Cardiovascular Disease, National Institute of Health and Medical Research, Toulouse, France; Melinda Alves, Institute for Metabolic and Cardiovascular Disease, National Institute of Health and Medical Research, Toulouse, France; Audrey Casemayou, Institute for Metabolic and Cardiovascular Disease, National Institute of Health and Medical Research, Toulouse, France; Guylène Feuillet, Institute for Metabolic and Cardiovascular Disease, National Institute of Health and Medical Research, Toulouse, France; Marie Buléon, Institute for Metabolic and Cardiovascular Disease, National Institute of Health and Medical Research, Toulouse, France; Manon Brunet, Institute for Metabolic and Cardiovascular Disease, National Institute of Health and Medical Research, Toulouse, France; Stéphane Gazut, Université Paris-Saclay, CEA, Palaiseau, France; Alberto Ortiz, Instituto de Investigación Sanitaria-Fundación Jiménez Díaz-Universidad Autónoma de Madrid, Madrid, Spain; Justyna Siwy, Mosaiques Diagnostics, Hannover, Germany; Jochen Metzger, Mosaiques Diagnostics, Hannover, Germany; Harald Mischak, Mosaiques Diagnostics, Hannover, Germany.

## Supplementary Material

sfae091_Supplemental_File

## Data Availability

Data are available upon reasonable request by email to the corresponding author.
