# Supplementary material for: Performances of acute kidney injury biomarkers vary according to sex
Source: Clin Kidney J. 2024 Mar 27;17(5):sfae091. doi: 10.1093/ckj/sfae091 (PMC11062024; doi:10.1093/ckj/sfae091)
Supplement: sfae091_Supplemental_File [file sfae091_supplemental_file.docx]

**Supplementary Table 1:** Odds ratio (confidence interval 95% and p-value) of AKI204 peptide signature, urinary NGAL or NephroCheck predicting acute kidney injury after adjustment on age, baseline estimated glomerular filtration rate, previous cardiac surgery status, duration of cardiac bypass and per-operative transfusion of red blood cells).

|  | **Males** | **Females** |
| --- | --- | --- |
| **AKI204 (score)** | 2.8 [2.2;3.6] p<0.0001 | 6.6 [3.8;11.5] p<0.0001 |
| **Urinary NGAL (μg/g)** | 1.001 [1;1.002] p=0.06 | 1.004 [1.001;1.008] p=0.007 |
| **Nephrocheck (>0.3)** | 1.91 [1.2;3.1] p=0.006 | 2.85 [1.04;7.8] p=0.05 |
